# Telomerase increasing compound protects hippocampal neurons from amyloid beta toxicity by enhancing the expression of neurotrophins and plasticity related genes

**DOI:** 10.1038/s41598-019-54741-7

**Published:** 2019-12-02

**Authors:** Natalie Baruch-Eliyahu, Vladislav Rud, Alex Braiman, Esther Priel

**Affiliations:** The Shraga Segal Department of Microbiology, Immunology & Genetics, Faculty of Health Sciences, Ben-Gurion University of the Negev, Beer-Sheva, Israel

**Keywords:** Molecular biology, Neuroscience

## Abstract

The telomerase reverse transcriptase protein, TERT, is expressed in the adult brain and its exogenic expression protects neurons from oxidative stress and from the cytotoxicity of amyloid beta (Aβ). We previously showed that telomerase increasing compounds (AGS) protected neurons from oxidative stress. Therefore, we suggest that increasing TERT by AGS may protect neurons from the Aβ-induced neurotoxicity by influencing genes and factors that participate in neuronal survival and plasticity. Here we used a primary hippocampal cell culture exposed to aggregated Aβ and hippocampi from adult mice. AGS treatment transiently increased *TERT* gene expression in hippocampal primary cell cultures in the presence or absence of Aβ and protected neurons from Aβ induced neuronal degradation. An increase in the expression of Growth associated protein 43 *(GAP43*), and Feminizing locus on X-3 genes (*NeuN*), in the presence or absence of Aβ, and Synaptophysin (*SYP*) in the presence of Aβ was observed. *GAP43, NeuN, SYP*, Neurotrophic factors (NGF, BDNF), beta-catenin and *cyclin-D1* expression were increased in the hippocampus of AGS treated mice. This data suggests that increasing TERT by pharmaceutical compounds partially exerts its neuroprotective effect by enhancing the expression of neurotrophic factors and neuronal plasticity genes in a mechanism that involved Wnt/beta-catenin pathway.

## Introduction

Telomerase is a reverse transcriptase protein that is best known for its telomere maintenance functions in dividing cells. Telomeres serve as substrates for telomerase that adds repeated sequences of hexa-nucleotides to the ends of chromosomes, thus maintaining telomere length^1,2^. The catalytic core of telomerase is composed of the RNA subunit TERC (Telomerase RNA Component) and the catalytic protein subunit TERT (Telomerase Reverse Transcriptase). Telomerase is active during embryonic development and its activity is repressed during embryonic differentiation, but remains active in germ line, stem cells and activated lymphocytes. The enzyme is reactivated in 90% of all cancers^2^.

Besides its role in maintaining telomere length, it was recently shown that the TERT protein has a variety of functions both *in vitro* and *in vivo* which are distinct from its canonical role of telomere extension. In the nucleus, TERT is involved in gene regulation, chromatin organization and DNA-damage response^3–5^. It is also shuttled from the nucleus to the mitochondria upon oxidative stress, where it decreases levels of ROS, DNA damage and apoptosis, and improves mitochondrial membrane potential, respiration and complex I activity^3,4,6,7^. Others and we demonstrated the expression of TERT, gene and protein, and telomerase activity in adult mouse brain^8–11^ and the presence of additional alternative TERC with an anti-oxidative stress activity^8^. *In vitro* studies on adult neurons and human brain tissue were also conducted demonstrating the non-canonical functions of TERT^6,12^. TERT shuttled from the nucleus to the mitochondria upon oxidative stress, in cultivated neurons and in the hippocampal neurons of AD brains^6^. The mitochondrially localized TERT decreases levels of ROS, DNA damage and apoptosis and neurons lacking TERT display an increased level of oxidative species and an increase in cellular oxidative damage^6^.

Alzheimer’s disease (AD) is a progressive and irreversible neurodegenerative disorder that is characterized by cognitive impairment, memory loss and characteristic pathological changes in the brain. The pathophysiology of the disease is complex and involves several neurotransmitter systems and pathophysiologic processes^13^. There are three hallmarks of AD: amyloid-beta (Aβ) plaques, neurofibrillary tangles, and neuronal cell death. These pathologies are evident in specific, vulnerable brain areas and the hippocampus is one of the earliest to be affected^13–15^. Although there is a debate to whether the deposition of Aβ plaques or neurofibrillary tangles of TAU are responsible for the initiation of the disease, we chose to base our model on the Aβ hypothesis. But it is likely that no single hypothesis will be able to account for all the underlying aspects of the disease process^14^. The possible neuroprotective effect of TERT from Aβ induced cytotoxicity was shown by exogenic expression of *TERT* gene in neurons^16^. Since genetic manipulations have severe limitations, we suggest that pharmaceutical increase of telomerase may show significant advantages. We previously synthesized novel tri-aryl compounds designated AGS and showed that these compounds increased *TERT* gene and TERT protein expression and telomerase activity in a time and dose-dependent manner and protected cells from oxidative stress both *in vivo* and *in vitro*^11,17,18^. This protection was demonstrated when AGS treatment of SOD1 transgenic mice that developed Amyotrophic Lateral Sclerosis has delayed the onset and progression of the disease and increased motor neuron survival in the mouse spinal cord^11^. To elucidate the mechanism by which telomerase increasing compounds exert their neuroprotective effect it is important to understand their effect on neurotrophins and neuronal plasticity associated genes and related signal transduction pathways such as Wnt.

Neurotrophic factors such as the Brain-Derived Neurotrophic Factor (BDNF) and the Neuronal Growth Factors (NGF) are crucial for processes such as growth, survival and differentiation of neurons from embryonic to adult stages^19^. NGF is mainly involved in the process of neuronal growth, plays a crucial role in the survival and maintenance of neurons, and neurons lacking NGF can undergo apoptosis^20^. While BDNF plays a similar role to that of NGF in respect of survival of neurons, it also takes part in functions such as differentiation of neurons, and synaptogenesis. In the Central Nervous System (CNS), BDNF is expressed in areas vital for learning such as the hippocampi, and the basal fore-brain^21^.

The Wnt signaling pathway is a group of highly conserved pathways that participate in processes such as gene transcription^22^, cell proliferation, cell migration, axes formation, and many others^23,24^. Wnt plays an important role in the brain and nervous system. Deficits in the pathway have been associated to impaired development^25^ and neurological disorders such as AD^26^. The importance of the Wnt signaling pathway in neurodegenerative disorders may lie in its role in processes such as neurogenesis and regulation of synaptic transmission and plasticity^26^. The functional importance of the Wnt signaling pathway in neuronal growth and plasticity may be due to the expression of neurotrophins such as BDNF, and NGF since their expression is regulated by Wnt^27,28^. A positive reinforcement loop between TERT and Wnt exists in which TERT can function as an activator of the Wnt signaling pathway^29^, while simultaneously being up-regulated by WNT signaling^30^.

In this study, we increased *TERT* gene expression using our novel AGS compounds. In a primary hippocampal cell culture that was exposed to aggregates of Aβ (an *ex vivo* model for AD) we found that increasing TERT by AGS offers a significant neuroprotective effect from the Aβ induced neuronal degradation. Treatment with the telomerase increasing compounds significantly enhanced the expression of genes involved in neuronal plasticity both in the *in-vitro* model of AD and *in vivo* in the hippocampus of AGS treated mice. In addition, a significant increase in the expression of BDNF (gene and protein) was demonstrated both *in vitro* and *in vivo* and the activation of the Wnt signaling *in vivo* by AGS treatment is established.

## Materials and Methods

### Animals

Neonatal ICR mice (1–2 days old) were used to prepare primary hippocampal cell cultures.

Adult ICR mouse hippocampi (6 weeks) were used for *in vivo* experiments.

The animal experimentation ethics committee at Ben-Gurion University approved all animal procedures. (IL-07-06-14, IL-11-09-2018B). All the experiments were performed in accordance with relevant guidelines and regulations.

### Preparation of primary cell cultures

Brains were quickly removed from the skull, and placed in a petri dish with HBSS + HEPES solution (20 mM, pH-7.4) on ice. The hippocampi were removed from the brains and cut into pieces. The tissues were placed in a protease solution that was pre-incubated in 37 °C for 15 min containing: 20 mM HBSS + HEPES solution (pH-7.4), 100 mM CaCl_2_, 50 mM EDTA (pH-7.4), 20 mg Cysteine crystals and 100 units/5 ml papain, in a final volume of 4 ml. The protease solution was shaken gently for 20 min at room temperature. After 20 min cells were centrifuged, the protease solution was removed and a medium containing: 92% Neurobasal Medium, 5% Fetal Bovine Serum, 2% B27, 1% Glutamate and 2 µl of Gentamicin was added.

### Plating of cells

Cells were suspended and plated. For staining, cells were plated in 24 wells plates at 0.25 × 10^5^ cells per well containing cover slips. For RNA and protein extractions, cells were plated in 6 wells plates at 4 × 10^5^ cell per well. The plates were pre-incubated for 1 hour with Poly-D-Lysine (PDL). PDL was removed, washed twice with water and once with the medium. After 24 hours of incubation at 37 °C, medium was removed and replaced with a medium containing: 97% Neurobasal Medium, 2% B27 and 1% Glutamate. The culture was maintained in the incubator for 14 days before further treatments and analysis.

### Preparation of aggregated Aβ

Aggregated Aβ (GL Biochem (Shanghai) Ltd.) was prepared as previously described^31,32^ briefly: the peptide was dissolved in hexafluoroisopropanol (HFIP) to 1 mM. HFIP was removed and dried under vacuum and the peptide was re-suspended in DMSO to 5 mM. For oligomeric conditions, F-12 Culture Media was added to bring the peptide to a final concentration of 100 µM. The experiments were conducted with concentrations of 5 µM and 10 µM Aβ that were prepared with cell medium.

### Preparation and injection of AGS

For cell culture treatment, as previously described^11,18^, AGS compounds were dissolved by DMSO and diluted with the cell medium to a final concentration of 20, 50, 100, and 200 nM.

For animal treatment, as previously described^11^ A single S.C. injection of 6 mg/ Kg was administered (n = 7 ICR mice per group). AGS stock solution in DMSO was freshly prepared for each experiment and 1 µl AGS/DMSO mixture in 100 μl PBS or an equivalent amount of the vehicle 1 µl DMSO in 100 μl PBS were administrated by subcutaneous injection (S.C) into the mouse neck.

### Immunofluorescent staining

#### Primary cell culture

Immunofluorescence analysis was performed with anti-β tubulin 3 (Sigma, Rehovot) mouse antibody as the first antibody, to stain microtubules and with anti-NeuN (Millipore) mouse antibody as the first antibody, to stain neuronal cell bodies. Cy3 anti mouse-immunoglobulin G (IgG; Jackson ImmunoResearch) was used as the second antibody in both cases. Nuclei were stained with 4′, 6-diamidino-2-phenylindole (DAPI).

Cells were fixed with cold (−20 °C) methanol and incubated overnight with either the anti-β tubulin 3Ab (1:250) or the anti-NeuN Ab (1:500) at 4 °C. Following a washing with PBS, the cells were incubated for 60 min at room temperature with secondary Ab Cy3 (1:50) and finally with DAPI (0.1 mg/ml) for 10 min at room temperature. β tubulin 3 staining was visualized using a confocal microscope (FluoView FV1000 Olympus, Tokyo, Japan).NeuN slides staining was visualized using a panoramic slide scanner (Panoramic Midi II scanner 3DHISTECH, Budapest, Hungary)

#### Mouse Hippocampal perfusion-fixed frozen sections

Were prepared as previously described^9,10^. The brain slices (5  μΜ) derived from vehicle (DMSO) and AGS-499 treated mice were subjected to immunofluorescence analysis using as first antibody: Guinea pig anti BDNF-antibody (1:100, # AGP-021, alomono labs, Israel) or anti-beta catenin antibody (1:100, β-Catenin (6B3) Rabbit mAb #9582, Cell Signaling technology, Danvers, MA, USA). For second antibody: Alexa –Fluor 635 conjugated anti guinea pig IgG (1:100, Life Technologies, #1736958) and cy2 conjugated anti-rabbit IgG (1:100, Jackson ImmunoResearch) were used respectively. We focus on the hippocampus region in the frozen brain slices. The IF slides were digitalized using the Panoramic Scanner 3DHISTECH0)

### Image analysis

NeuN stained slides were analyzed using the CellProfiler^TM^ software.

The amount of NeuN positive and DAPI positive cells was quantified separately using the IdentifyPrimaryObjects pipeline. The amount of NeuN positive cells was then divided by the amount of DAPI positive cells and normalized to 100 percent. NeuN positive cell size was determined using MeasureObjectSizeShape pipeline. The object size list was then processed using GraphPad Prism to obtain a distribution of cell size. Cells were then categorized into 3 size groups.

The IF slides of the hippocampal sections were analyzed by the CaseViewer 2.3 software. The auto- fluorescence button was applied and all the images were adjusted to the same parameters of: Black = 0, Gamma = 1.60, White = 65535.

### Real time PCR

RNA extract was prepared with “Bio Tri RNA” (Bio-Labs) kit per the manufacturer instructions.

cDNA was prepared using the qScript cDNA synthesis kit (QuantaBio).

Real time PCR was performed using the following primers:

#### Tert

Probe: 5′ - /56-FAM/CACTGCGTA/ZEN/TAGCACCTGTCACCAA/31ABkFQ/ - 3′

Forward: 5′ GACTTCTTCCTGCACTTCCTG 3′; Reverse: 5′ CTTGTTCTCCATGTCTCCGA 3′

#### β - Actin

Probe: 5′ - /56-FAM/CTGGCCTCA/ZEN/CTGTCCACCTTCC/31ABkFQ/ - 3′

Forward: 5′ GATTACTGCTCTGGCTCCTAG 3′; Reverse: 5′ GACTCATCGTACTCCTGCTTG 3′

For the TERT and beta actin genes, we used the PrimeTime Mini qPCR Assay (Integrated DNA Technologies) with the 7500 Real time PCR System (Applied BioSystems).

#### NeuN

Forward: 5′ CGGCGGAAACCTCCTCGG 3′; Reverse: 5′ TCAACGGGTTCAGCGTTCCC 3′

#### Synaptophysin

Forward: 5′ CTGCGTTAAAGGGGGCACTA 3′; Reverse: 5′ACAGCCACGGTGACAAAGAA3′

Growth Associated Protein 43 (GAP43)

Forward: 5′ AGATGGTGTCAAGCCGGAAG 3′; Reverse: 5′ CATCCTTCTCCTTGGCCTCG 3′

60 s acidic Ribosomal Protein P0 (RPLP0)

Forward: 5′ CCAGCAGGTGTTTGACAACG 3′; Reverse: 5′ TCCAGAAAGCGAGAGTGCAG 3′

#### Brain Derived Neurotrophic Factor (BDNF)

Forward: 5′ TCATACTTCGGTTGCATGAAGG 3′; Reverse: 5′ ACACCTGGGTAGGCCAAGTT 3′

#### Neuronal Growth Factor (NGF)

Forward: 5′ ACTGGACTAAACTTCAGCATTCC 3′; Reverse: 5′ GGGCAGCTATTGGTGCAGTA 3′

#### β-catenin

Forward: 5′GTGCTATCTGTCTGCTCTAGTA 3′; Reverse: 5′CTTCCTGTTTAGTTGCAGCATC 3′

#### Doublecortin (DCX)

Forward: 5′ TTCGTAGTTTTGAGCGTTGCT 3′; Reverse: 5′GAGGCAGGTTAATGTTGTCAG 3′

#### Sex Determining Region Y Box-2 (SOX-2)

Forward: 5′ AACCCCAAGATGCACAACTC 3′; Reverse: 5′ GCTTAGCCTCGTCGATGAAC3′

#### Cyclin D1

Forward: 5′AATCGTGGCCACCTGGATG 3′ Reverse: 5′CTTCAAGGGCTCCAGGGACA 3′

The RT-PCR for the above genes was performed using the perfeCTa SYBER Green Fast Mix for quantitative PCR (QuantaBio) with the 7500 Real time PCR System (Applied BioSystems).

The RQ for all the examined genes was calculated by the delta- delta CT method using beta actin or RPLP0 (ribosomal RNA) as the reference housekeeping genes. The data of each treatment (AGS and DMSO) was normalized to the untreated group to confirm that no significant effect of the vehicle treatment on the expression of genes was detected.

### Statistical analysis

Data was expressed as mean ± SEM. Significant differences were determined using one-way ANOVA followed by a posterior Bonferroni test for multiple comparisons or by Student’s T test (for 2 groups) provided by GraphPad Prism version 6.00 for Windows (GraphPad Software, San Diego, CA, USA).

## Results

### Telomerase increasing compound (AGS) enhanced *TERT e*xpression in primary hippocampal cell culture in a dose and time dependent manner and protected neurons from amyloid beta toxicity

The primary hippocampal cell cultures were stained with anti GFAP and anti-beta 3 tubulin antibodies and DAPI for nuclei. The results show that the culture consist of the various cell types of the hippocampus: it contained neurons stained by the beta3 tubulin and many astrocytes and astroglial cells stained by GFAP (Supl. 1) and the % of neurons in the culture was also estimated (20%) by NeuN staining as described later in the text.

**Figure 1:**
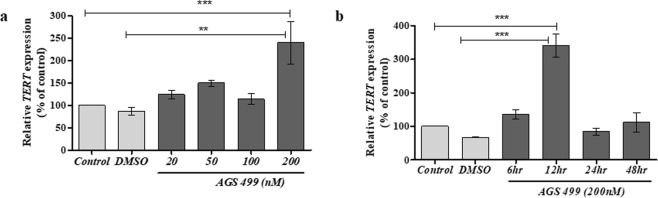
AGS increases *TERT* gene expression levels in a time and concentration dependent manner in a primary hippocampal cell culture. (**a**) Cultures were treated for 12 hr with AGS-499 at concentrations of 20 nM, 50 nM, 100 nM, and 200 nM or with its vehicle DMSO. (**b**) Cultures were treated with AGS-499 at a concentration of 200 nM for 6 hr, 12 hr, 24 hr, and 48 hr periods. RNA was extracted from cultures and converted to cDNA using a reverse transcriptase kit. Expression levels were quantified using RT-PCR and normalized to the untreated control (Control). The results are Mean ± SEM, n = 4 independent experiments), ANOVA test ***p < 0.001 ^**^p < 0.005.

**Figure 2:**
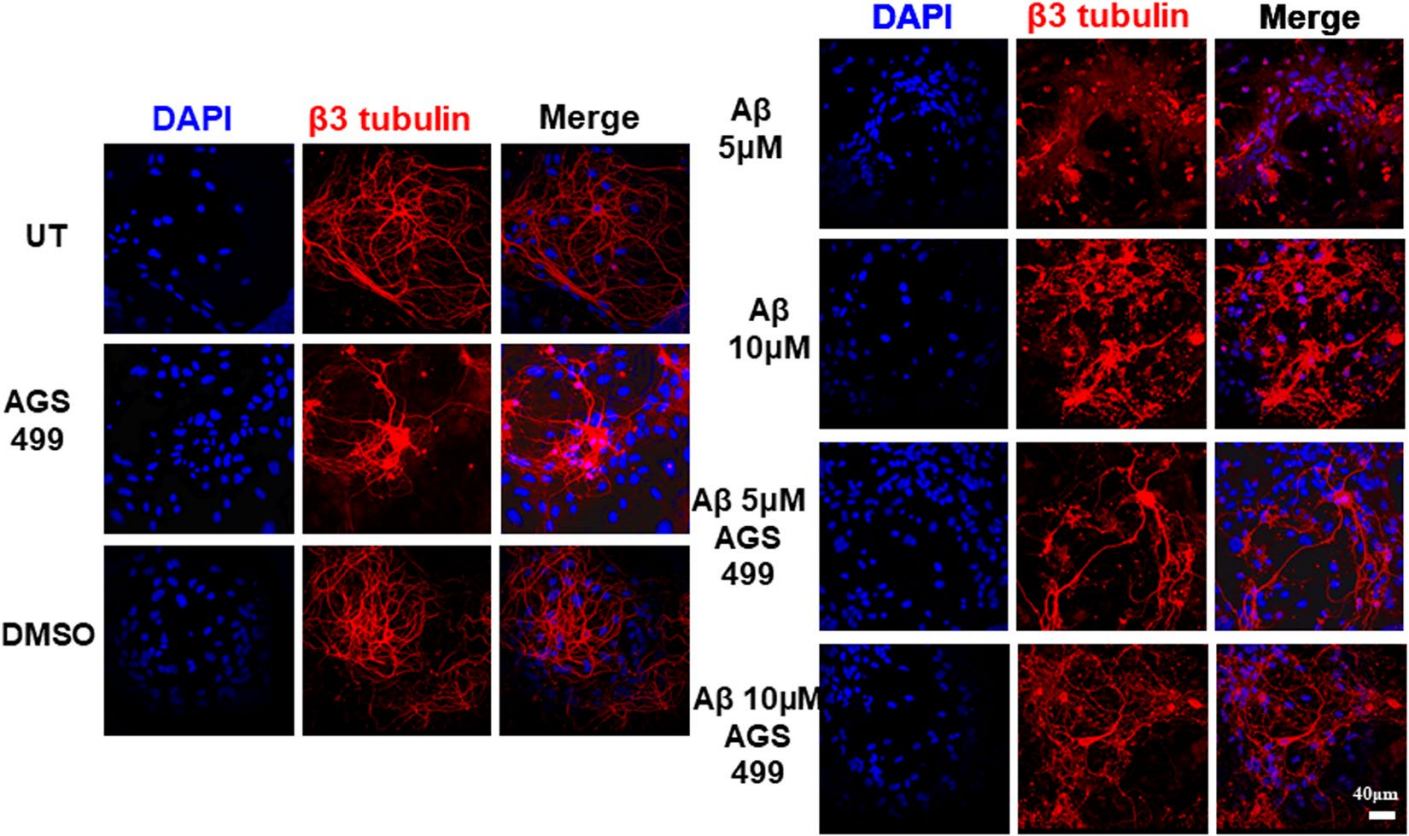
AGS Improves hippocampal neuron morphology and reduces Aβ induced neuronal degradation in primary hippocampal cell cultures. The cell culture was treated with/without oligomeric Aβ for 48 hrs and AGS-499 (or DMSO) was added to the culture medium every 24 hrs. Immunofluorescent staining using DAPI and anti β-III tubulin antibody after 48 hours of the various treatments was performed. Slides were visualized using confocal microscopy (FluoView FV1000 Olympus). Scale bar: 40 μm

The culture was treated with varying concentrations of AGS-499 from 20 nM to 200 nM for 12 hrs (Fig. 1a). An increase in *TERT* gene expression was observed up to 2.8-fold with 200 nM of AGS (compared to UT or vehicle treated cells). A transient increase in *TERT* gene expression (up to 5 fold, 12-hrs. post treatment) was detected in the AGS-499 treated primary hippocampal cell culture, which returns to its basal level 24 hrs post treatment (Fig. 1b). Primary hippocampal cell cultures were treated with oligomerized Aβ protein at 5 μM and 10  μM with and without AGS treatment (200 nM) for 48 hrs. The AGS compound (AGS-499) was administered every 24 hrs. The cells were subjected to an immunofluorescence procedure with anti-β3-tubulin antibodies (for staining of neurites) and with DAPI as a nuclear staining for all the cell types in the primary culture (Fig. 2). As can be seen, treatment with the Aβ protein at 5 μM and 10 μM caused significant neuronal degradation demonstrated by the fragmentation of axons and neurites and the presence of smaller cell bodies. The degradation effect of Aβ protein on neurons was visibly higher at 10 μM compared to 5 μM. In the AGS treated culture a significant visible increase in neuronal survival was observed which is demonstrated by the presence of neurons with long axons, neurites, and normal cell body (soma) size, suggesting that AGS treatments conferred partial protection of neurons from the Aβ induced damage at both 5 μM and 10 μM Aβ protein treatments.

### The expression of *Gap43* and *NeuN* genes is enhanced in primary hippocampal cell cultures treated with telomerase increasing compound in the presence or absence of Aβ

**Figure 3:**
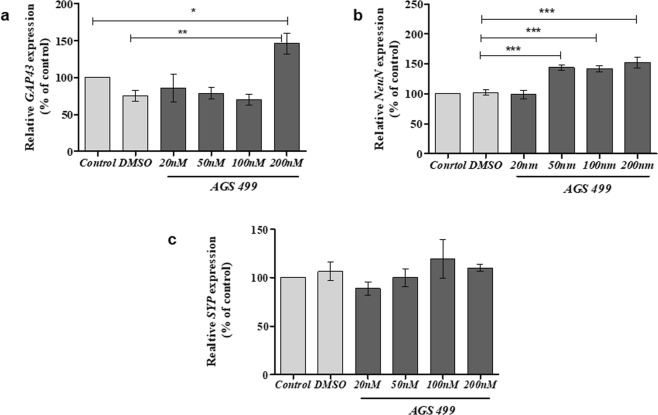
AGS increases *TERT* and neuronal gene expression levels in a primary hippocampal cell culture. Cultures were treated for 12 hr with AGS-499 at concentrations of 20 nM, 50 nM, 100 nM, and 200 nM or with its vehicle DMSO. RNA was extracted from cultures and genes Expression levels were quantified using RT-PCR and normalized to the untreated control (Control). (**a**) The effects of AGS-499 treatment on the expression of *GAP43* gene. (**b**) The effects of AGS-499 treatment on the expression of Synaptophysin gene. (**c**) The effects of AGS-499 treatment on the expression of *NeuN* gene. *The results are Mean* *±* *SEM, n* *=* 4 independent experiments), ANOVA test *p *<* 0.05, **p *<* *0.01*.

To elucidate the mechanism by which the telomerase increasing compound enhanced the survival of neurons in the presence of Aβ protein, we first examined the effect of AGS alone on the expression of neuronal genes that are involved in the morphology of neuronal cell body and axonal growth. The hippocampal cell culture was treated with and without various AGS-499 concentrations for 12 hrs. and the expression of the following genes was determined: *NeuN* for neuronal soma^33^, Growth Associated Protein 43 (*GAP43*) for axonal growth^34^, and Synpatophysin (*SYP)* a major synaptic protein p38 for synapses formation and quantification^35^. The results depicted in Fig. 3 demonstrate that AGS treatment significantly increased *Gap43* gene expression up to1.95 fold (Fig. 3a) *NeuN* gene expression up to1.5 fold (Fig. 3b) and no significant change in the expression of *SYP* gene (Fig. 3c) compared to untreated or vehicle treated cells.

**Figure 4:**
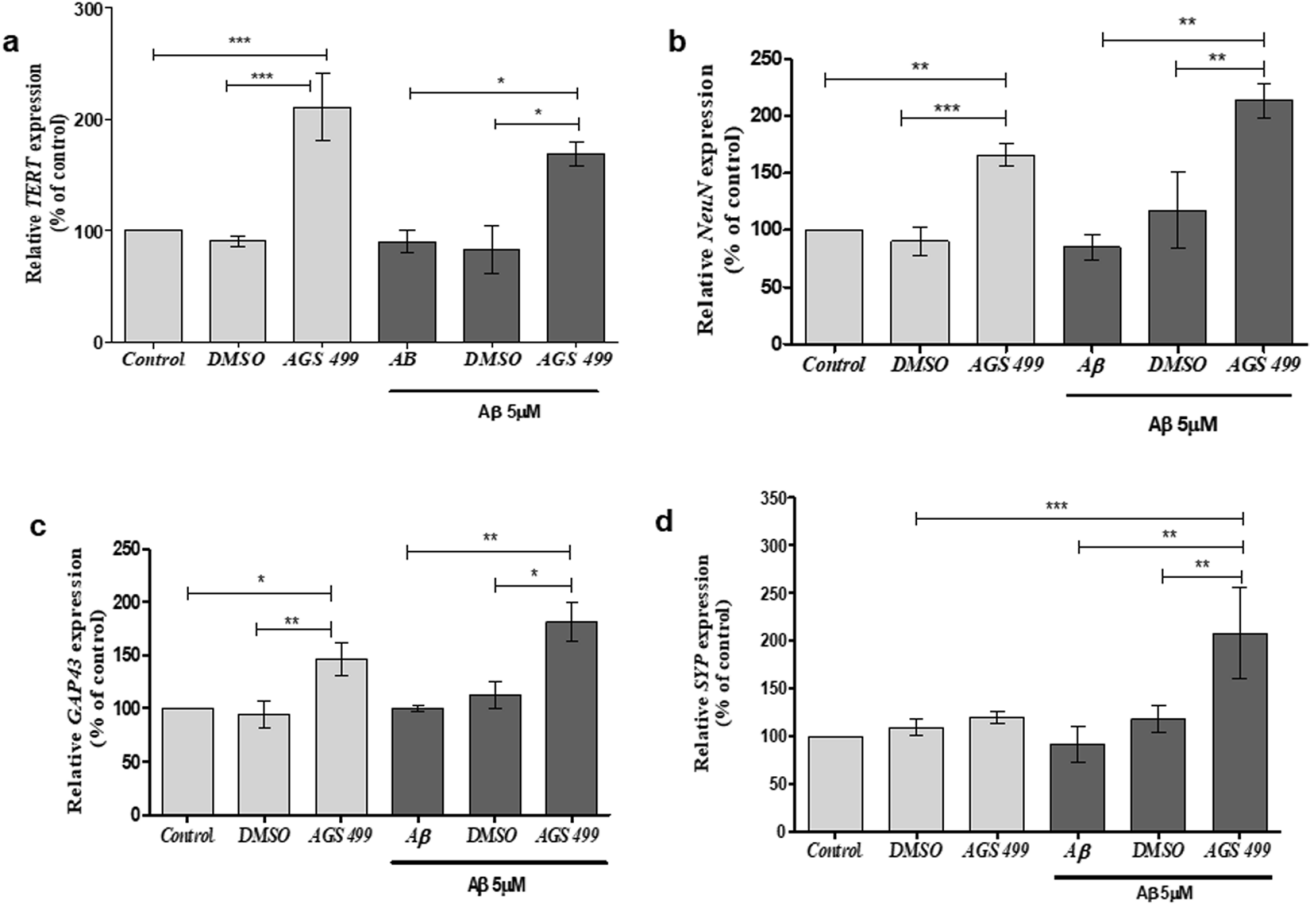
AGS increases *TERT* and neuronal gene expression levels in Aβ treated cultures. Cultures were treated for 36 hr. with AGS-499 (200 nM) alone, Aβ (5 µM) alone, and Aβ (5 µM) in combination with AGS-499 (200 nM) or its vehicle DMSO. AGS or DMSO treatments were renewed after 24 hr. Genes Expression levels were quantified using RT-PCR and normalized to the untreated control (Control). (**a**) Relative *TERT* gene expression in cultures (Mean ± SEM, n = 5 independent experiments), ANOVA test *p < 0.05, ***p < 0.001. (**b**) Relative *NeuN* gene expression in cultures (Mean ± SEM, n = 4 independent experiments), ANOVA test **p < 0.01, ***p < 0.001. (**c**) Relative *GAP43* gene expression in cultures (Mean ± SEM, n = 4 independent experiments), ANOVA test *p < 0.05, **p < 0.01. (**d**) Relative *SYP* gene expression in cultures (Mean ± SEM, n = 4 independent experiments), ANOVA test **p < 0.01, ***p < 0.001.

Next, we examined the effect of telomerase increasing compound on the expression of the aforementioned neuronal genes and the expression of *TERT* gene in hippocampal cells that were exposed to oligomers of Aβ protein. Cell cultures were incubated with 5 μM Aβ for 36 hrs, in the presence or absence of the AGS-499 compound, which was added to the cell culture every 24 hours. The results depicted in Fig. 4a revealed that *TERT* gene expression was not affected by the exposure of the cells to Aβ protein and the AGS-499 compound can increase the expression of *TERT* up to 2.3 -fold in the presence of Aβ protein. The expression of *NeuN* (Fig. 4b), *Gap43* (Fig. 4c) and *SYP* (Fig. 4d) genes were not significantly altered by Aβ protein treatment, but were significantly increased by AGS treatment in the presence of Aβ protein: up to 2-fold for *NeuN* (Fig. 4b), up to 1.5-fold for *Gap43* (Fig. 4c) and up to 2.5-fold for *SYP* (Fig. 4d) genes. It should be noted that AGS treatment increased the expression of *SYP* gene only in hippocampal cell culture that was exposed to Aβ protein (Fig. 4d), while the other neuronal genes *NeuN* and *GAP43* were also significantly increased in cell cultures that were treated with AGS-499 alone.

### The expression of neurotrophins in primary hippocampal cell cultures is increased following treatment with AGS compound

**Figure 5:**
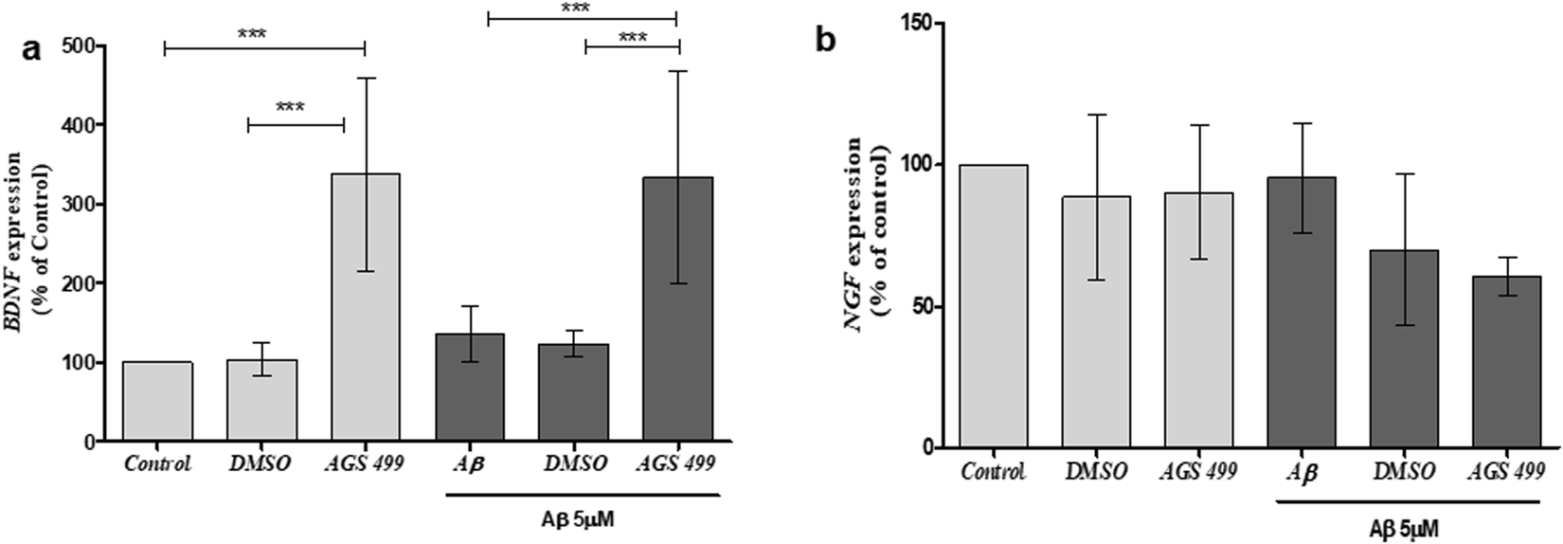
AGS increases *BDNF* but not *NGF* gene expression in both Aβ treated and untreated cultures. Cultures were treated for 36 hr. with AGS-499 (200 nM) alone, Aβ (5 µM) alone, and Aβ (5 µM) in combination with AGS-499 (200 nM) or with its vehicle DMSO. AGS or DMSO treatments were renewed after 24 hr. Expression levels were quantified using RT-PCR and normalized to the untreated control (Control). (**a**) Relative *BDNF* gene expression in cultures (Mean ± SEM, n = 5 independent experiments), ANOVA test ***p < 0.001. (**b**) Relative *NGF* gene expression in cultures (Mean ± SEM, n = 5 independent experiments), ANOVA test.

NGF and BDNF, the two main neurotrophins affect the expression of neuronal genes such as *GAP43* and *SYP*^36,37^ and demonstrate AD related neuroprotective effects^38,39^. Therefore we examined the effect of AGS treatment on the expression of *NGF*, and *BDNF* genes. Cell cultures were incubated with 5 μM Aβ for 36hrs with and without AGS-499 compound that was added to the cell culture every 24 hours. The results in Fig. 5 show that AGS-499 significantly increased the expression of *BDNF* gene (up to 3-fold) in the presence of Aβ oligomers (Fig. 5a) while eliciting no effect on *NGF* gene expression (Fig. 5b).

### AGS treatment increased the relative amount of neurons in the primary hippocampal culture and protected against Aβ induced neuronal loss

**Figure 6:**
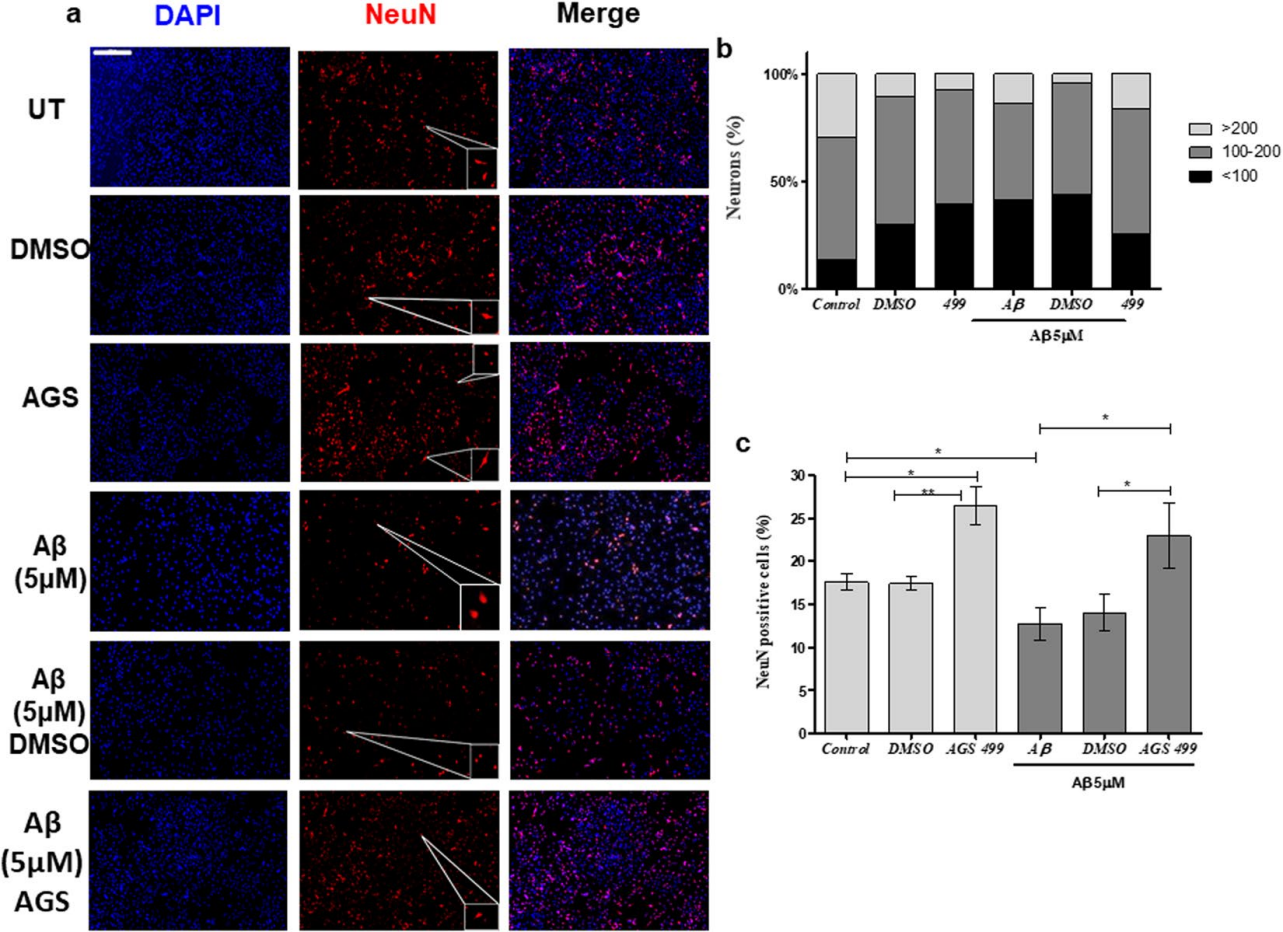
AGS increases the relative amount of NeuN positive cells, and protects from neurodegeneration. (**a**) Immunofluorescent staining using DAPI and NeuN after 48 hours of the indicated various treatments compared to an untreated control. Slides were visualized using a panoramic slide scanner (Panoramic Midi II scanner 3DHISTECH, Budapest, Hungary). Scale bar: 500 μm.The enlarged images were obtained using the sniping tool software for the selected area in the pictures (**b**) Cell size distribution. Cell size was determined using CellProfiler software. Cell sizes were divided into 3 groups of arbitrary size. Cells with a size smaller than 100 pixels (<100), a size of between 100 and 200 pixels (100–200) and cells larger than 200 pixels (>200). (**c**) Relative amount of NeuN positive cells. Normalized to total amount of DAPI stained cells. (Mean ± SEM, n = 5), ANOVA test *p < 0.05, **p < 0.01.

The increase in the expression of both *NeuN* and *BDNF* genes in AGS treated cultures, together with the role of BDNF in the process of neurogenesis^40,41^ may suggest that the AGS treatment increased the amount of NeuN expressing cells in the hippocampal cell culture. To examine this possibility, cell cultures were incubated with Aβ (5 μM) for 48 hours, with and without the AGS-499 compound that was added to the cell culture every 24 hours, and stained for NeuN protein and DAPI. The stained cultures were quantified for both the size of NeuN positive cells, and the relative quantity of NeuN positive cells was calculated as percentage of all the DAPI stained cells in culture (Fig. 6b,c). The results demonstrate that the hippocampal cell culture contained up to 20% NeuN positive cells. Treatment with Aβ alone significantly decreased the percentage of NeuN positive cells, and the remaining NeuN positive cells also decreased in size. However, AGS treatments, both alone and in combination with Aβ, significantly increased the percent of NeuN positive cells (Up to 1.35-fold and 1.8-fold compared to the untreated cultures and the Aβ treated cultures respectively). In addition, an increase in the size of the NeuN expressing cells in the presence of Αβ oligomers following AGS treatment, compared to cultures treated with Aβ alone was detected (Fig. 6b)

### *In Vivo*: The expression of *TERT* and neuronal genes increased in hippocampi of AGS treated mice

**Figure 7:**
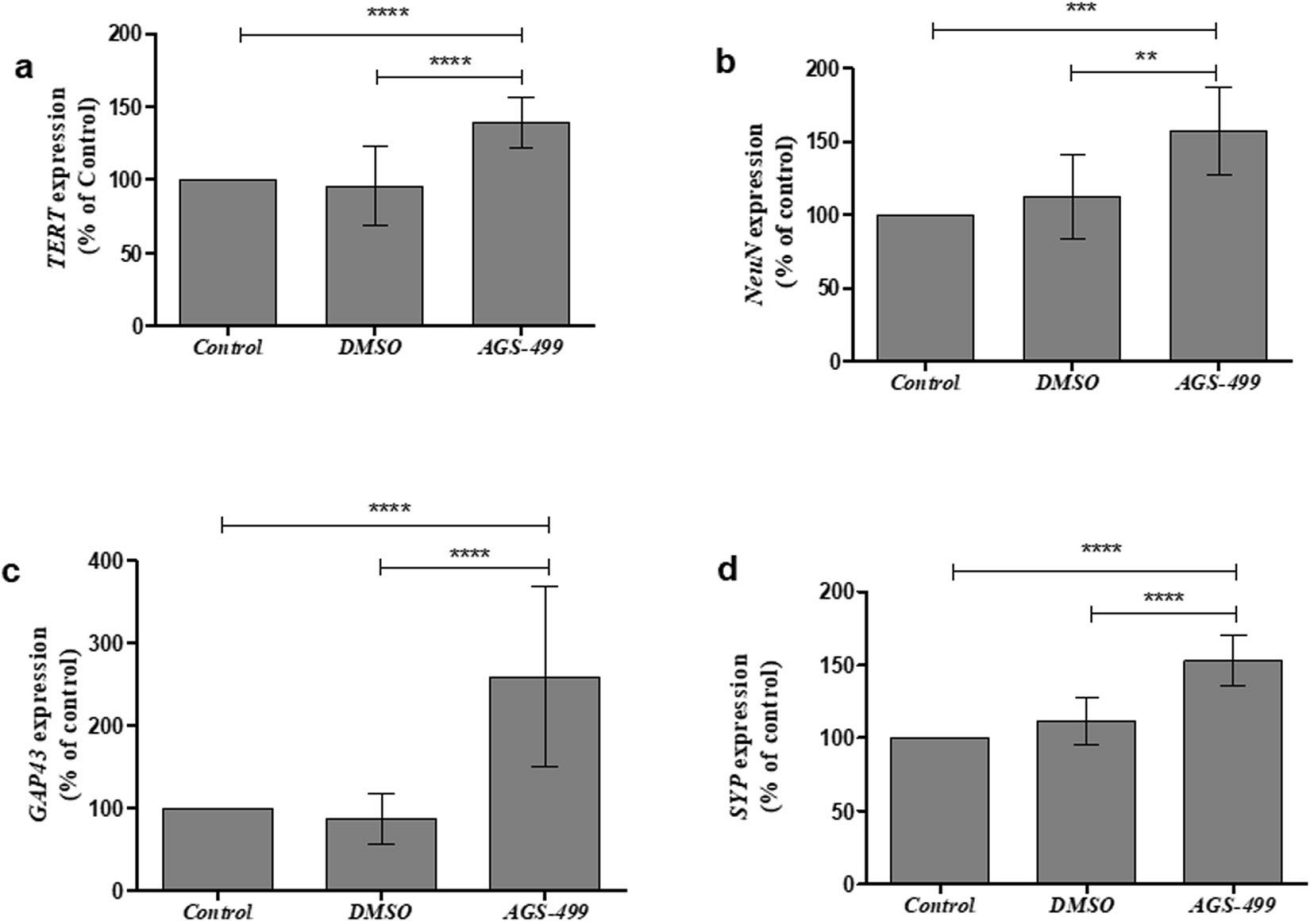
AGS increases the expression of TERT and neuronal genes *in vivo*. Mice were injected subcutaneously with AGS-499 in a concentration of 6 mg /kg of body mass. After 12 hr., mice were sacrificed. Expression levels were quantified using RT-PCR and normalized to the untreated control (Control). (**a**) Relative *TERT* gene expression *in-vivo*, in hippocampi (Mean ± SEM, n = 7 mice per group), ANOVA test ****p < 0.0001. (**b**) Relative *NeuN* gene expression *in-vivo*, in hippocampi (Mean ± SEM, n = 7 mice per group), ANOVA test **p < 0.01, ***p < 0.001. (**c**) Relative *GAP43* gene expression *in-vivo*, in hippocampi (Mean ± SEM, n = 7 mice per group), ANOVA test ****p < 0.0001. (**d**) Relative *SYP* gene expression *in-vivo*, in hippocampi (Mean ± SEM, n = 7 mice per group), ANOVA test ****p < 0.0001.

**Figure 8:**
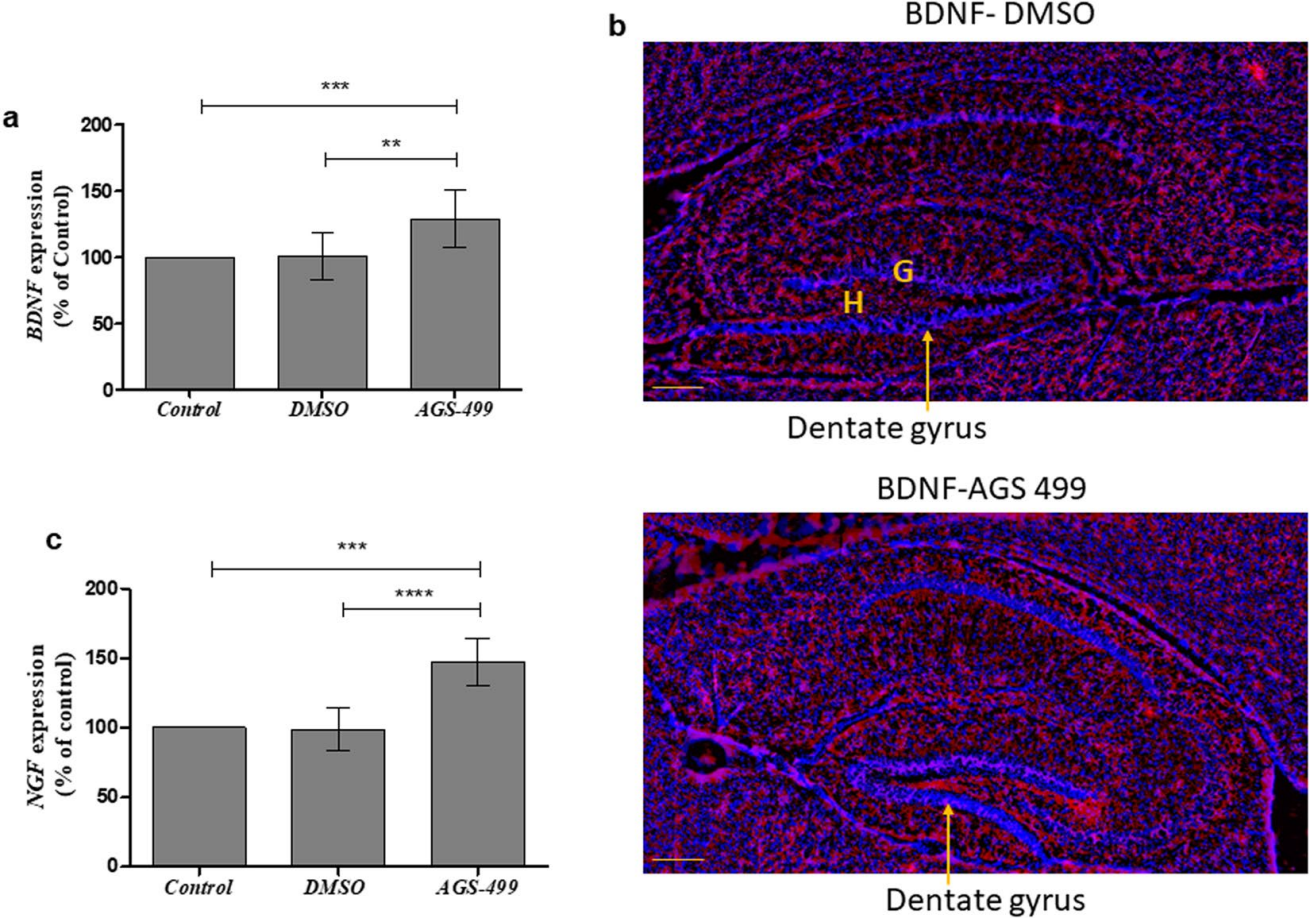
AGS increases neurotrophins expression levels *in vivo*. Mice were injected subcutaneously with AGS-499 in a concentration of 6 mg per kg of body mass. After 12 hr., mice were sacrificed. (**a**) Expression levels were quantified using RT-PCR and normalized to the untreated control (Control) and compared to vehicle treated mice. The results are Mean ± SEM, n = 7 mice per group), ANOVA test **p < 0.01, ***p < 0.001. **(b**)Immunofluorescence analysis of perfusion fixed frozen hippocampal sections was performed using anti BDNF antibody (red) DAPI staining for nucleus (blue).G-granule layer, H-hilus. Scale bare 200 μm. **(c)** Relative *NGF* gene expression *in-vivo*, in hippocampi (Mean ± SEM, n = 7 mice per group), ANOVA test ***p < 0.001, ****p < 0.0001.

The significant effect of the AGS compound on the expression of neuronal genes in the primary hippocampal cell cultures was tested *in vivo*. Six-week-old ICR mice were injected with the compound as previously described^11^ and the expression of *TERT, NeuN*, *Gap43*, and *SYP* genes in the hippocampus was determined. A significant increase in the expression of these genes was detected: *TERT* up to 1.5-fold (Fig. 7a), *NeuN* up to 1.5-fold (7b), *GAP43* up to 2.5-fold (7c), and *SYP* up to 1.5-fold (7d), suggesting that a single treatment with AGS compound increased the expression of these genes in the mouse hippocampus *in vivo*. The effect of the AGS treatment on the expression of neurotrophins in the hippocampus *in vivo* was also examined and the results depicted in Fig. 8 revealed that the expression of *BDNF* gene was significantly increased up to 1.5-fold in AGS-499 treated mice compared to untreated or vehicle treated mice (Fig. 8a). Immunofluorescence analysis of hippocampal perfusion-fixed frozen sections using specific anti BDNF antibody, revealed an increase in BDNF staining in the hippocampus (the dentate gyrus granule layers the hilus and the interneurons outlines) in AGS treated mice compared to vehicle treated mice (Fig. 8b). *NGF* gene expression was also significantly increased in AGS0499 treated mice compared to vehicle treated mice (Fig. 8c)

**Figure 9:**
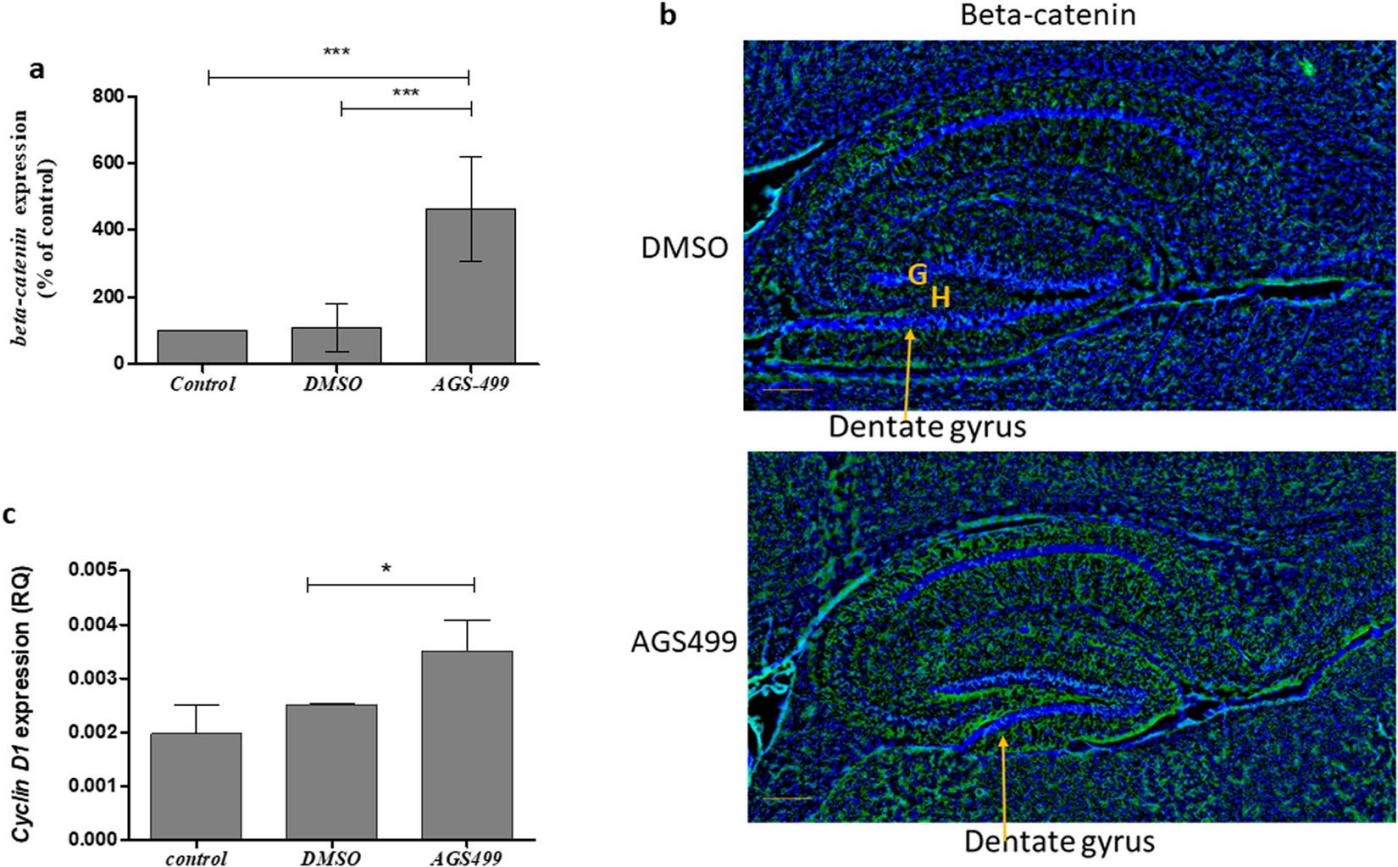
AGS increases beta-catenin and *cyclin D1* expression. Mice were injected subcutaneously with AGS-499 in a concentration of 6 mg per kg of body mass. After 12 hr., mice were sacrificed. Expression levels were quantified using RT-PCR and normalized to the untreated control (Control) and compared to vehicle treated mice. (**a**) Relative β-catenin gene expression *in-vivo*, in hippocampi (Mean ± SEM, n = 7 mice per group), ANOVA test ***p < 0.001. (**b**) Immunofluorescence analysis of perfusion fixed frozen hippocampal sections was performed using anti beta-catenin antibodies(green) and DAPI staining for nucleus (blue). G-granule layer, H-hilus. Scale bare 200 μm. (**c**) Relative *cyclin D1* gene expression *in-vivo*, in hippocampi (Mean ± SEM, n = 7 mice per group), Student’s T test *p < 0.05

Telomerase also regulates the transcriptional activity of the β-catenin dependent Wnt signaling pathway^29^. Using our previously injected mice, we determined the expression of β-catenin gene and protein as a measure of WNT signal activity as was previously demonstrated^42^. The results depicted in Fig. 9a show that AGS-499 treated mice expressed elevated levels of β-catenin gene - of up to 4-fold in the hippocampus, compared to untreated or vehicle treated mice. Immunohistofluoresence analysis of hippocampal perfusion- fixed frozen sections using specific anti beta-catenin antibody, revealed a significant increase of beta-catenin staining in the hippocampus of AGS treated mice compared to untreated mice (Fig. 9b).

To confirm that the Wnt/beta catenin pathway is active we examined the expression of *cyclin D1* gene that its level is regulated by the Wnt-beta catenin signaling^43^.Indeed the expression of *cyclin D1* in the hippocampus of AGS treated mice significantly increased compared to vehicle treated mice (Fig. 9c).

**Figure 10:**
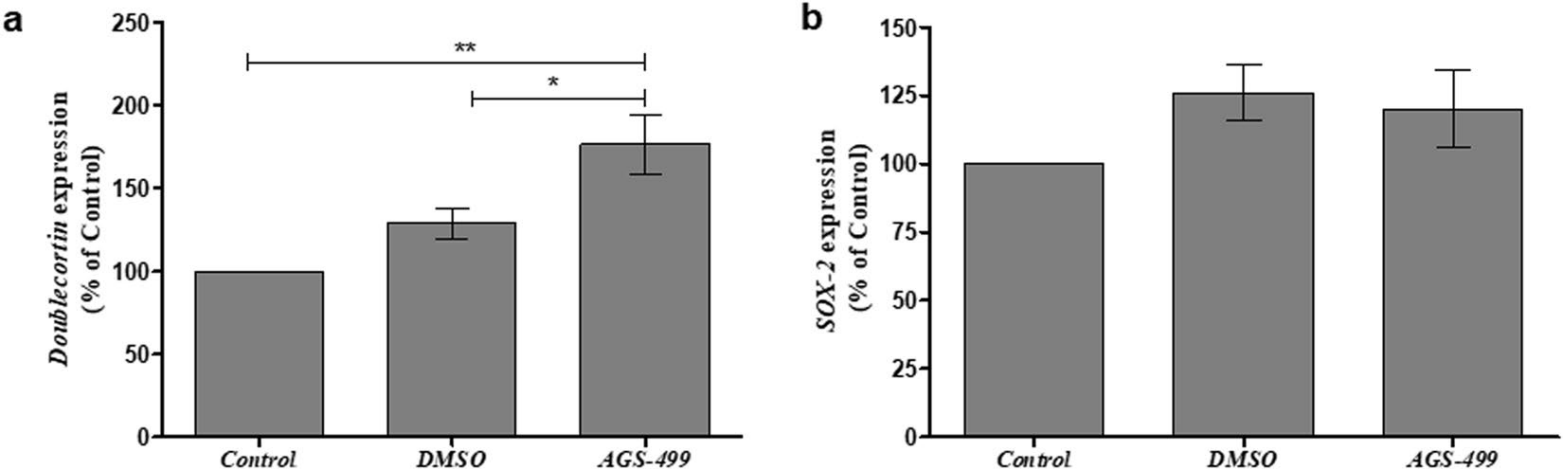
AGS increases *Doublecortin* gene but not *SOX-2* gene expression *in vivo*. Mice were injected subcutaneously with AGS-499 in a concentration of 6 mg per kg of body mass. After 12 hr., mice were sacrificed. Expression levels were quantified using RT-PCR and normalized to the untreated control and compare to vehicle. (**a**) Relative *DCX* gene expression (Mean ± SEM, n = 6 mice per group), ANOVA test *p < 0.05, **p < 0.01. (**b**) Relative *SOX-2* gene expression (Mean ± SEM, n = 6 mice per group), ANOVA test.

AGS treatment of the primary hippocampal cell culture in the presence or absence of Aβ significantly increased the relative amount of NeuN expressing cells. This increase could be due to a neurogenesis process. We examined this possibility *in vivo* by investigating the expression of genes known to be expressed in various stages of the neuro-differentiation pathway: Doublecortin (DCX), and Sex Determining Region Y box-2 (SOX-2). DCX is a known marker for neurogenesis^44,45^. Its expression starts in type-2b progenitor cells and persists until an immature post-mitotic neuronal cell is produced. SOX-2 is an early stem cell marker expressed in Neuronal Stem Cells (NSCs) and early type 2-a progenitor cells. The expression of DCX and SOX-2 is mutually exclusive^46^. The results depicted in Fig. 10 show that AGS injection caused a significant increase of up to 1.5-fold in the expression of *DCX* gene (Fig. 10a), while no change in *SOX-2* gene has been detected (Fig. 10b).

## Discussion

In this study, we examined the effect of increasing *TERT* gene expression by a pharmaceutical compound on the protection of neurons from the Αβ-induced toxicity. This assumption was based on several studies; it was previously shown that the reduction of TERT expression results in enhanced sensitivity of neurons to the common neurotoxic proteins expressed in AD pathology^16^. In addition, it was suggested that overexpression of telomerase exerted some neuroprotective effects from Aβ in AD models^47^. However, genetic manipulation as a therapeutic strategy possesses several limitations and pharmaceutical treatments are therefore preferred. Indeed, we previously showed that novel tri-aryl compounds designated AGS transiently increased *TERT g*ene and TERT protein expression and telomerase activity both *in vivo* and *in vitro* in a time and dose dependent manner^11,17,18^. These compounds can cross the BBB and transiently increase telomerase activity and TERT expression in the brain^11^. Treatment of SOD1 Tg mice with these compounds delayed the onset and progression of ALS and increased the survival of motor neurons in the mouse spinal cord^11^. Here we used a common in *vitro* model for AD - primary hippocampal cell cultures exposed to oligomeric Aβ. First, we demonstrated that *TERT* gene expression is transiently increased in these cultures following AGS treatment. The exposure of the primary hippocampal cell culture to cytotoxic concentrations of oligomeric Aβ did not significantly affect *TERT* gene expression, while treatment with AGS in the presence of Aβ has increased *TERT* gene expression. These results demonstrate that the AGS compound can increase the expression of *TERT* in the hippocampal cells also in the presence of Aβ aggregates.

The aggregated exogenic Aβ possesses neurotoxic properties, and it affects the morphology of neurons. Untreated neuronal cultures display normal cellular morphology with: intact axons, connections to neighboring cells via neurites, and proper soma size; while treatment of the culture with Aβ, both at 5 μM and 10 μM, caused axonal degradation, synaptic loss and shrinkage of the cell body. Whereas treatment with AGS-499 alone did not affect the neurons in the culture, it significantly conferred partial protection from the cytotoxicity induced by Aβ at both concentrations (5 and 10 μM).

It should be noted that these Aβ concentrations are significantly higher by about 1000 fold than the physiological concentration of the peptide in the brain, which is in the range of pico and nano molars^48,49^.

The neuroprotective effect of AGS 499 is demonstrated here by the morphology of the neurons (normal size of soma body, long axons and neurites). To confirm the neuroprotective effect of AGS by molecular tools we investigated the effect of AGS treatment on the expression of genes that are associated with neuronal soma and neuronal plasticity. Interestingly, we found that treatment of the primary hippocampal cell cultures with telomerase increasing compound alone significantly increased the expression of *NeuN* and *GAP43* genes but not the *SYP* gene. This data demonstrates a correlation between the increase in the expression of *TERT* gene and the enhancement in the expression of *NeuN* and *GAP43* genes. It was previously suggested that TERT may act as a transcriptional factor for certain genes^3–5^, however it is not yet clear whether this is a direct or indirect effect of TERT on the expression of *NeuN* and *Gap43* genes. Exposure of the primary hippocampal cell culture to cytotoxic aggregates of Aβ slightly but not significantly reduced the expression of *NeuN, GAP43*, and *SYP* genes, but when AGS treatment was applied to the Aβ treated cultures, a significant increase in the expression of these genes was observed. The specificity of NeuN expression in neurons of the central nervous system is well established^33^, but its role is not fully understood. The protein is expressed only in post-mitotic neuronal cells and has been implied, among other things, in RNA editing^50^. The increase in NeuN expression in AGS treated hippocampal neurons in the presence or absence of oligomeric Aβ might be due to an increase in the expression levels within existing mature neurons, or is the consequence of maturation of neurons from neuronal precursor cells, or even due to both processes occurring simultaneously. However, our data shows that the number of NeuN positive cells has increased in the hippocampal cell culture by 30% following AGS treatment. Exposure of the culture to Aβ decreased the NeuN positive cells by 40% while treatment with AGS compounds dramatically increased the number of NeuN expressing cells by 183%. In addition, treatment with telomerase increasing compounds gives rise to a morphological new population of neurons; these neurons, while also expressing NeuN, are smaller and round in shape compared to their more common counterparts. The round shape of the cells indicates a lack of a typical neuronal structure known as the axon hillock^51^. These small, NeuN expressing cells that lack structures found in mature neurons, could be a population of young, newly formed, post-mitotic neurons. Therefore, it is possible that treatment of the primary hippocampal cell culture by the telomerase increasing compound induced neurogenesis, a possibility that was previously demonstrated in the brain of telomerase-reactivated mice^52^. GAP43 is highly expressed in neuronal growth cones during development and axonal regeneration^34^. The increase in *GAP43* following AGS treatment of either Aβ-treated or untreated hippocampal cell culture may suggest an increase in the growth process of axons. In addition, Gap 43 was identified as a rapidly transported axonal protein that is highly upregulated after sciatic nerve injury. It is localized to growth cones associated with neuropil areas and is highly expressed during nervous system developmental and regenerative axon growth^53^. Therefore, one may assume that the increase of its expression by AGS treatment in damaged axons points toward its involvement in the neuroprotective processes of the telomerase increasing compound.

Although the expression of SYP is known to be limited to synaptic vesicles, its function is not. It is however used as marker to quantify synapses^35^. Synaptic alterations are one of the earliest events in AD and it was found that oligomeric Aβ disrupts synapses^54,55^.

Although AGS-499 treatment alone did not increase *SYP* gene expression it did however enhance its expression in hippocampal cultures that were exposed to Aβ, thus suggesting that in neurons damaged by Aβ-induced cytotoxicity, treatment with telomerase increasing compound, AGS-499, may restore the synapses formation by increasing the expression of the *SYP* gene.

All together our data show a significant increase in the expression of *NeuN, GAP43* and *SYP* genes, in the hippocampal cell cultures that were exposed to Aβ and treated with AGS, suggesting that in the *in vitro* AD model, the increase in their expression is part of the neuroprotective mechanism of the telomerase increasing compound. We injected AGS-499 into adult mice to examine whether the enhancement of the expression of neuronal genes, involved in neuronal plasticity and neuronal growth, is a unique phenomenon observed only in hippocampal cell culture derived from newborn mice, or rather can occur *in vivo* in adult mice. We previously showed that this compound can cross the BBB and can increase TERT expression in the various regions of the brain of adult mice^11^. Here we found that a single injection of AGS-499 increased *TERT* gene expression in the hippocampus of both male and female mice. We also showed that a single injection of AGS-499 significantly increased the expression of *NeuN*, *GAP 43* and *SYP* genes *in vivo* in the mouse hippocampus. This strongly indicates that the effects of telomerase increasing compound are not only an *in vitro* phenomenon but also occur *in vivo*. It should be noted that unlike the *in vitro* data in which *SYP* gene expression was not affected by AGS-499 alone, this compound significantly increased *SYP* gene expression in the mouse hippocampus. The biological effects of the increased expression of *NeuN, Gap43* and *SYP* genes in the mouse brain following AGS treatment are now under investigation in our group.

Since it was suggested that the expression of NeuN, GAP 43 and SYP might be regulated by neurotrophins^36,56,57^, we examined whether treatments with telomerase increasing compound affect the expression of neurotrophins - thus promoting processes such as neuronal development and survival^58^.

Out of the many members in this family, we focused on the main two: BDNF and NGF. In an *in vitro* primary culture, treatments with AGS-499 significantly increased the expression of *BDNF* gene but not *NGF* gene while *in vivo* the expression of both *BDNF* and *NGF* genes and BDNF protein were elevated following AGS treatment. BDNF and NGF play an important role in similar neuronal related pathways such as differentiation, maturation, growth, survival of existing neurons, and neurogenesis^58–60^. Since BDNF supports the survival of existing neurons, its increased expression in AGS-499 treated cultures may partially explain the neuroprotective effects of telomerase increasing compounds. In addition to their role in neuronal survival, both BDNF and NGF are important modulators of neuronal development and plasticity and have been shown to increase the expression of both GAP43 and SYP^36,56^. BDNF and NGF play also an important role in the process of formation of new neurons, also known as neurogenesis^58^. This role has led us to hypothesize that the increase in NeuN (gene and protein) expression, which correlates to the increase in both BDNF and NGF following AGS treatments, stems from the formation of new neuronal cells. To further demonstrate the possibility of induced neurogenesis, in AGS treated mice, we used two mutually exclusive markers of the various differentiation stages of the neuronal cell – SOX-2 and DCX. While SOX-2 is expressed only in embryonic stem cells, DCX appears in late neuronal progenitor cells and its expression persists until the early stages of post-mitotic neurons^44–46^. Indeed an increase in *DCX* gene expression was observed in the hippocampus of AGS treated mice suggesting an increase in neuronal differentiation, while the lack of increase in *SOX-2* gene expression indicates that no increase in stem cell proliferation was observed. Therefore, it is possible that the increase in the expression of BDNF and NGF by the telomerase increasing compounds result in the enhancement of the expression of neuronal plasticity related genes and induced neurogenesis, and thus contribute to the mode of action of the AGS compound as neuroprotective agent.

We previously showed that AGS compounds exerted their neuroprotective effects in a telomerase dependent manner^11^. To elucidate the mechanism by which increasing TERT expression might affect the expression of neurotrophins we examined the Wnt signaling pathway. Wnt signaling pathway is known to be activated by TERT^29,61^ and BDNF and NGF are known to be regulated by Wnt signaling^28,62^. We found that β-catenin expression (gene and protein) is increased in the hippocampus of AGS treated mice. Moreover *Cyclin D1* gene that is known to be regulated by the Wnt-beta catenin signaling^43^ was also increased in AGS treated mice compared to vehicle treated mice. These data indicates the activation of the Wnt-beta catenin signaling pathway^63^ by treatment with the AGS compound.

**Figure 11:**
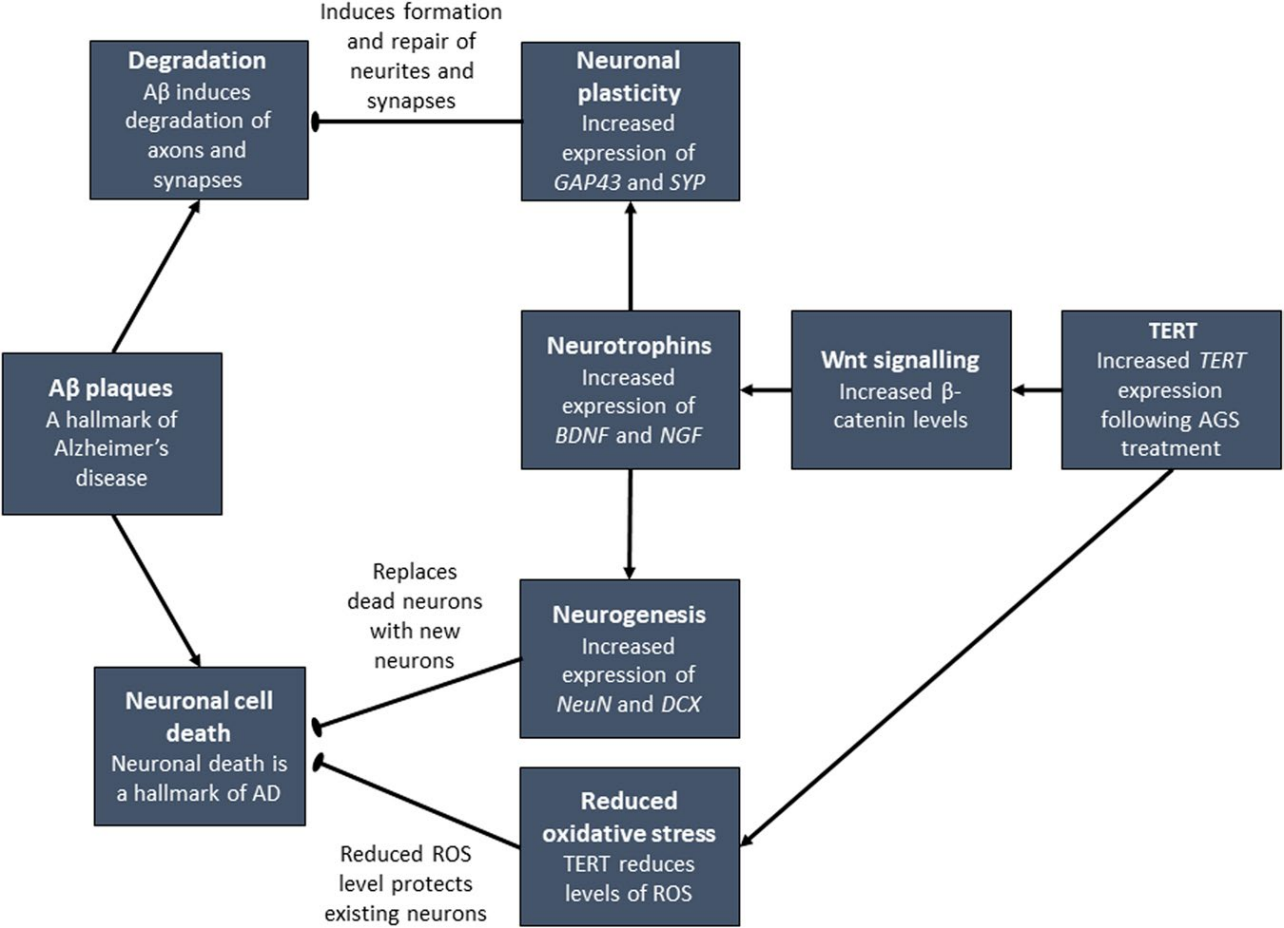
A proposed model for the neuroprotective effects of telomerase increasing compound AGS from the Aβ cytotoxicity in an *in-vitro* AD model. Increasing TERT expression in hippocampal cells activates the Wnt signaling pathway which upregulates the expression of neurotrophins that increase the expression of neuronal plasticity genes and therefore confer a partial neuroprotective effect from the Aβ induced cytotoxicity.

In conclusion, we propose, as summarized in Fig. 11, that increasing TERT expression in the hippocampus using a pharmaceutical compound partially exerts neuroprotective effect via activation of the Wnt signaling pathway. This activation leads to an increased expression of neurotrophins that in turn enhances the expression of neuronal growth and plasticity genes. This provides damaged neurons, such as those exposed to the cytotoxicity of Aβ, the ability to overcome its cytotoxic effects. In addition, the non-canonical roles of TERT, such as an anti-apoptotic enzyme and its mitochondrial protective function from oxidative stress are also part of the AGS neuroprotective effects as we previously demonstrated^11^. Our data suggest the possibility that the telomerase increasing compound may show some beneficial effects in AD and this is now under investigation using a mouse model for AD.

## Supplementary information

Supplementary information
