# Supplementary material for: Telomerase increasing compound protects hippocampal neurons from amyloid beta toxicity by enhancing the expression of neurotrophins and plasticity related genes
Source: Sci Rep. 2019 Dec 2;9:18118. doi: 10.1038/s41598-019-54741-7 (PMC6889131; doi:10.1038/s41598-019-54741-7)
Supplement: Supplementary file 1 — Supplementary information [file 41598_2019_54741_MOESM1_ESM.pdf]

Supplementary materials

Telomerase increasing compound protects hippocampal neurons from amyloid beta toxicity by enhancing the expression of neurotrophins and plasticity related genes

Natalie Baruch-Eliyahu[#], Vladislav Rud[#], Alex Braiman, Esther Priel[*]

Supl 1: Cells present in the primary hippocampal cell culture

We examined the major types of cells present in the Primary hippocampal cell culture from neonatal mice (1-2 days old) that was prepared as described in the "Materials and Methods" section. The cells in the culture were stained with anti-β tubulin 3 (Sigma, Rehovot) mouse antibody as the 1st antibody, to stain neuronal microtubules and with anti- glial fibrillary protein GFAP (Millipore) rabbit antibody as the 1st antibody, to stain the astrocytes and other non-neuronal cells in the culture. Cy3 anti mouse-immunoglobulin G (IgG; Jackson ImmunoResearch) and Cy2 anti-rabbit IgG (Jackson ImmunoResearch) were respectively used as the 2nd antibody. Nuclei were stained with 4', 6-diamidino-2-phenylindole (DAPI). The results presented in SUPl1 show that the primary hippocampal cell culture used in our experiments contained neurons stained by the beta3 tubulin and many astrocytes and astroglial cells stained by GFAP.

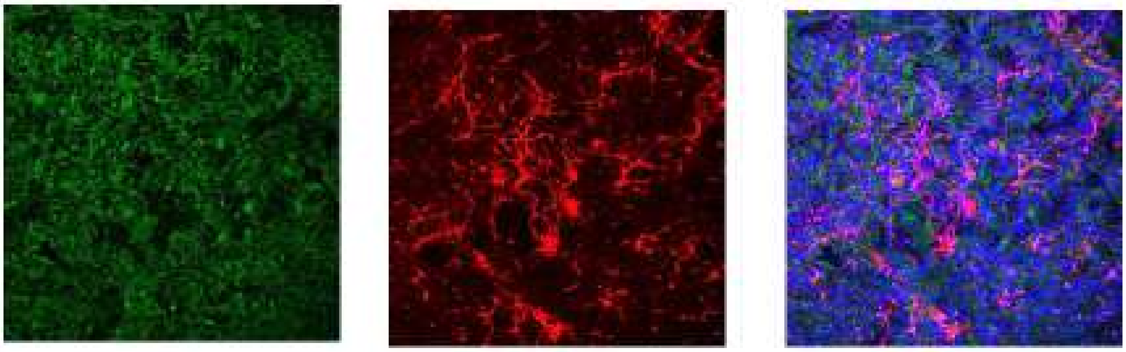

GFAP staining — β3 tubulin staining — Merge- GFAP, β3 tubulin and DAPI staining
